# A Case Report on the Pitfalls in Diagnosing Left Atrial Appendage Thrombus: Lessons From a False-Positive Transesophageal Echocardiography (TEE) in Mitral Valve Surgery

**DOI:** 10.7759/cureus.109438

**Published:** 2026-05-22

**Authors:** Nicole Bianca S Libiran, Edwin Becher, Makoto Kataoka

**Affiliations:** 1 Department of Anesthesiology, Indiana University School of Medicine, Indianapolis, USA

**Keywords:** 3d transesophageal echocardiography, cardiac computed tomography (cct), echocardiographic artifact, false-positive transesophageal echocardiography, left atrial appendage thrombus, mitral valve surgery, reverberation artifact, spontaneous echocardiographic contrast, transesophageal echocardiography (tee), ultrasound enhancing agents

## Abstract

A 47-year-old female with severe mitral stenosis underwent open mitral valve replacement. Preoperative and intraoperative transesophageal echocardiography (TEE) suggested a left atrial appendage thrombus, but no thrombus was found when the atrium was opened. While TEE is the gold standard for left atrial appendage thrombus diagnosis, its accuracy can be affected by artifacts, as well as normal anatomical structures, such as the coumadin ridge or pectinate muscle trabeculations, which may mimic thrombus, and by spontaneous echogenic contrast. Adjunct imaging techniques, including 3D TEE, left atrial appendage outflow velocity, and contrast-enhanced imaging, can improve accuracy. Cardiac CT is also a valuable alternative. In this case, the mass may have resulted from blood stasis rather than a true thrombus, highlighting the diagnostic challenges in such scenarios.

## Introduction

Left atrial appendage thrombus (LAAT) is a clinically significant concern in patients undergoing mitral valve surgery due to its association with thromboembolic complications. Transesophageal echocardiography (TEE) is considered the gold standard for preoperative LAAT detection; however, it has critical limitations. Artifacts and normal anatomical structures can mimic thrombus, leading to false-positive interpretations, surgical delays, and unnecessary interventions. Complex cardiac anatomy, such as prominent pectinate muscles, multilobed appendages, and para-cardiac or epicardial fat pads, may appear as hypodense masses adjacent to the LAA [1,2]. Spontaneous echocardiographic contrast (SEC), characterized by dense echogenic swirling blood in low-flow states, can also mimic thrombus [3]. Additionally, reverberation artifacts, particularly near the Coumadin ridge, a variably prominent structure between the left upper pulmonary vein and the LAA, may create or obscure thrombus-like appearances [4]. Here, we report a case in which preoperative and intraoperative TEE suggested LAAT, which was later disproven by direct surgical inspection, and we discuss the limitations of TEE as well as the potential role of adjunct imaging techniques.

## Case presentation

**Figure 1:**
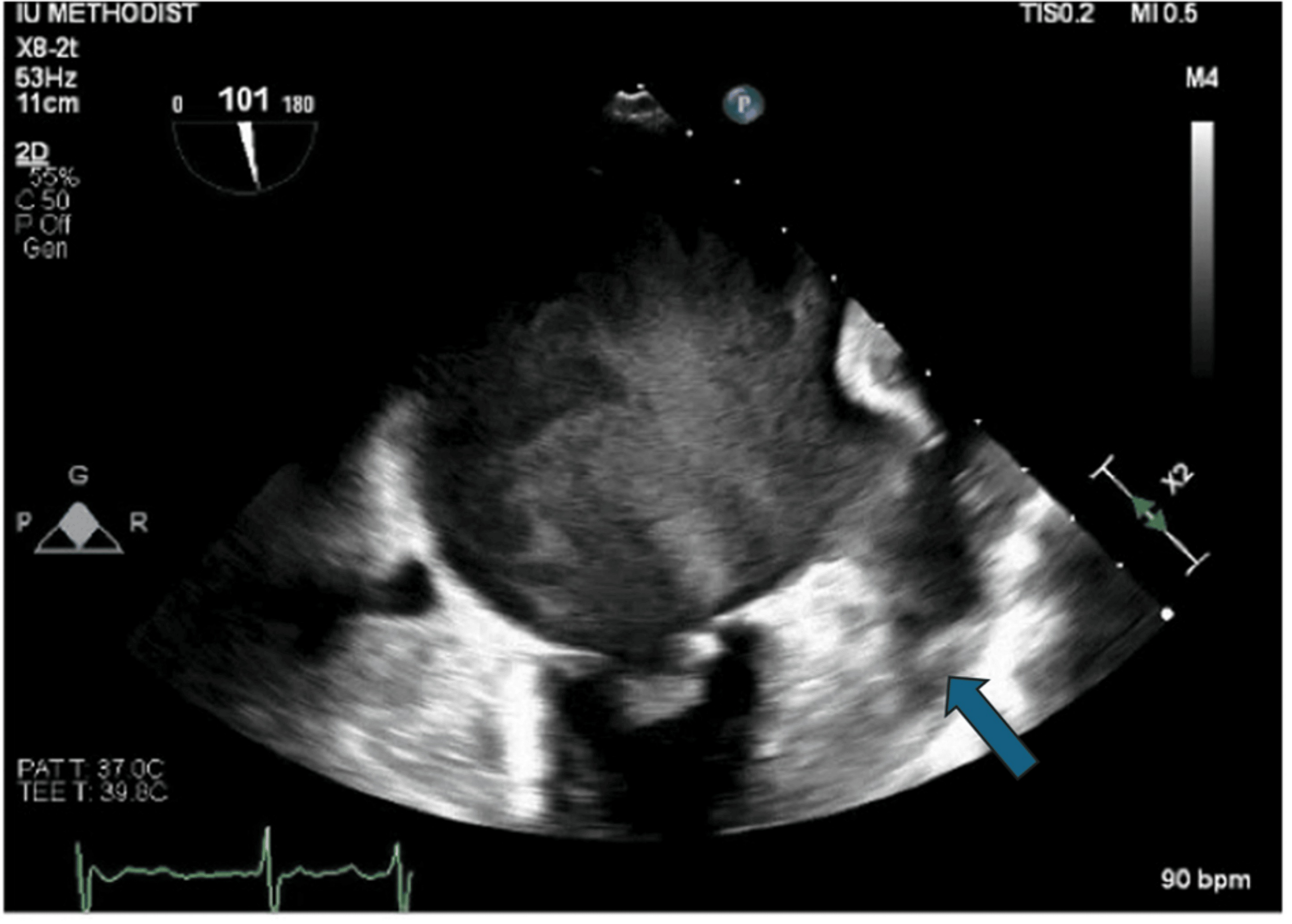
Transesophageal echocardiography image demonstrating an echodense mass within the left atrial appendage (blue arrow), concerning for thrombus

A 47-year-old female with a medical history significant for human immunodeficiency virus, permanent atrial fibrillation, seizure disorder, hypothyroidism, leukopenia, anxiety, and rheumatic mitral valve disease was scheduled for elective mitral valve replacement (MVR) for severe rheumatic mitral stenosis. Preoperative TEE revealed severe mitral stenosis with thickened anterior and posterior valve leaflets, a left ventricular ejection fraction (LVEF) of 50%, and an echodense mass within the LAA suggestive of thrombus (Figure 1). No regional wall motion abnormalities were demonstrated on preoperative TEE. Multiplanar TEE imaging and three-dimensional TEE reconstruction further demonstrated the suspicious echodensity concerning for possible thrombus formation (Videos 1-5).

**Video 1:** Transesophageal echocardiography (TEE) video in the omniplane 90-degree view. The red arrow indicates an echodense structure within the left atrial appendage, concerning for possible thrombus.

**Video 2:** Transesophageal echocardiography (TEE) video in biplane mode, displaying simultaneous orthogonal views at 0 and 90 degrees. The red arrow highlights an echodense area within the left atrial appendage, concerning for possible thrombus.

**Video 3:** Transesophageal echocardiography (TEE) video in the omniplane 135-degree view. The red arrow points to an echodense structure within the left atrial appendage, concerning for possible thrombus.

**Video 4:** Transesophageal echocardiography (TEE) video in biplane mode displaying simultaneous views at 45 and 135 degrees. The red arrow indicates an echodense area within the left atrial appendage, concerning for possible thrombus.

**Video 5:** Three-dimensional transesophageal echocardiography (3D TEE) reconstruction of the left atrial appendage, providing enhanced spatial visualization for the assessment of possible thrombus

**Figure 2:**
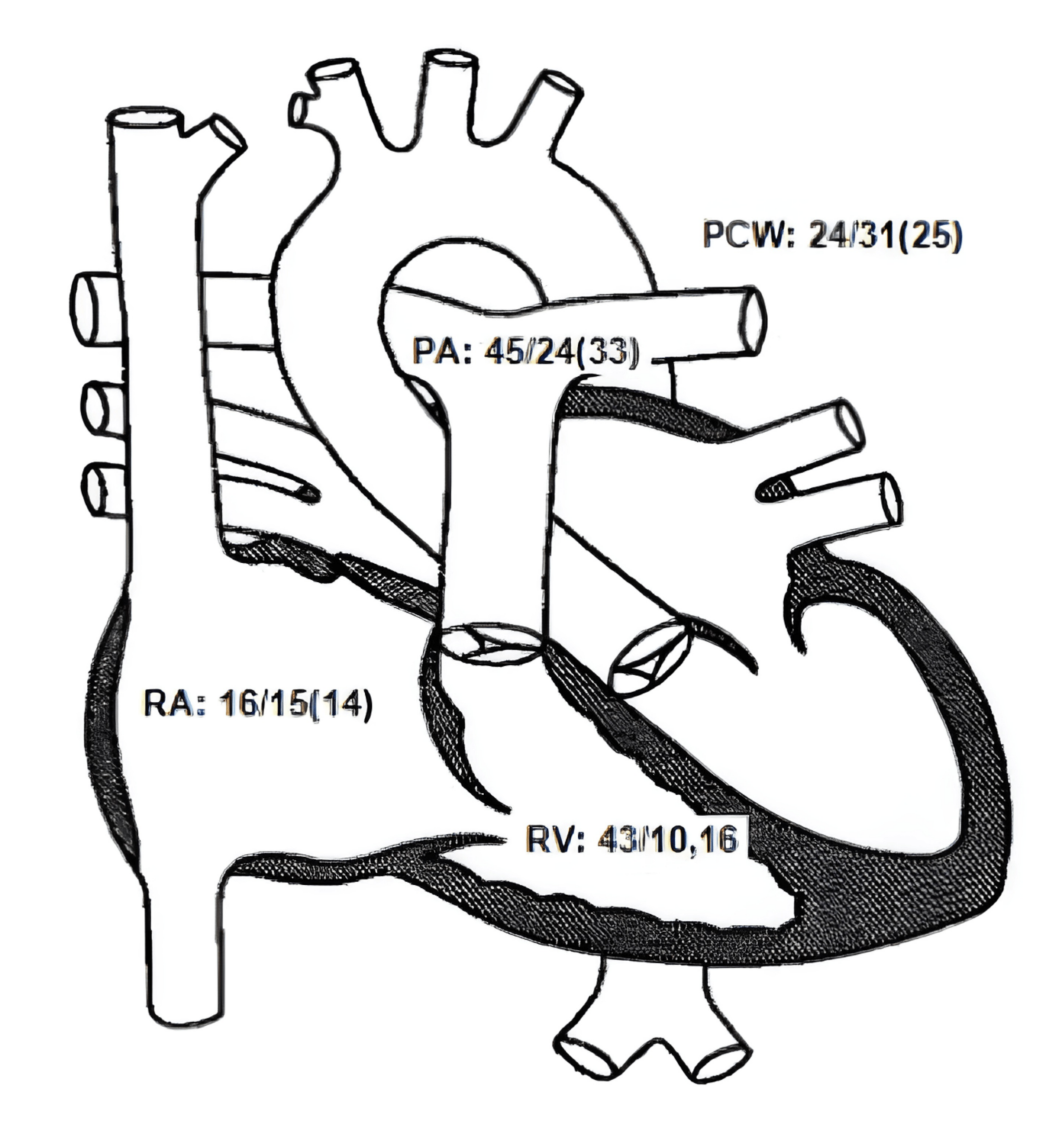
Hemodynamic pressures obtained from right heart catheterization, demonstrating elevated pulmonary artery (PA) pressures (45/24 mmHg, mean 33) Image credit: Original screenshot of a clinical right heart catheterization report generated from the hospital’s diagnostic software.

**Figure 3:**
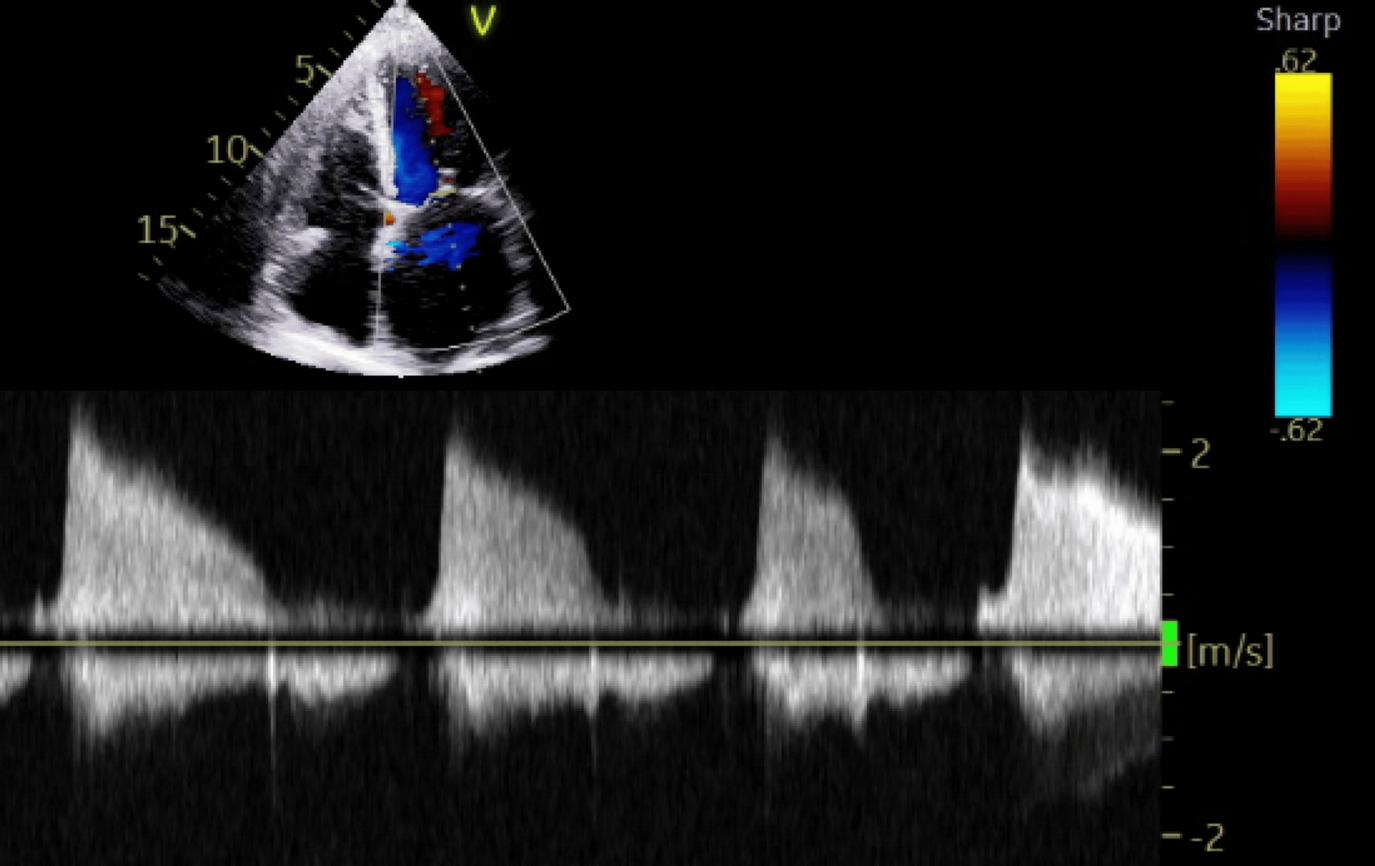
Transthoracic echocardiography in the apical four-chamber view demonstrating a mean transmitral pressure gradient of 9 mmHg

Prior cardiac catheterization showed no obstructive coronary artery disease (Videos 6, 7) and moderate pulmonary hypertension (Figure 2). Transthoracic echocardiography (TTE) in the apical four-chamber view (Video 8) showed features of rheumatic mitral valve stenosis, including leaflet thickening, calcification, and restricted mobility. The mean transmitral pressure gradient was measured at 9 mmHg (Figure 3), consistent with moderate mitral stenosis. Additional views revealed the classic “fish-mouth” mitral valve appearance (Video 9) and the “hockey stick” configuration of the anterior mitral leaflet during diastole (Video 10), both characteristic of rheumatic mitral stenosis.

**Video 6:** Coronary angiography demonstrating a normal right coronary artery (RCA) without evidence of obstructive coronary artery disease

**Video 7:** Coronary angiography demonstrating a normal left coronary artery (LAD) and left circumflex artery (LCX) without evidence of obstructive coronary artery disease

**Video 8:** Two-dimensional transthoracic echocardiography (TTE) in the apical four-chamber view, demonstrating features consistent with rheumatic mitral valve stenosis

**Video 9:** Parasternal short-axis view at the mitral valve level demonstrating the classic “fish-mouth” appearance of the mitral orifice, consistent with commissural fusion seen in severe mitral stenosis

**Video 10:** Parasternal long-axis view demonstrating the “hockey stick” appearance of the anterior mitral leaflet, characteristic of rheumatic mitral stenosis

Intraoperatively, no gross sludge or organized thrombotic material was identified or aspirated. Postoperatively, the patient was extubated on postoperative day (POD) 0 and remained hemodynamically stable. Vasoactive infusions were discontinued, and a heparin infusion was initiated for anticoagulation of the mechanical prosthetic valve by POD 1. Scheduled neurologic checks were consistently performed in the ICU. The patient experienced no neurologic complications from POD 0 through POD 5 and was discharged on POD 5. The absence of neurologic symptoms in this patient makes it unlikely that a thrombus, if truly present, embolized during the surgery. Notably, the patient has experienced no embolic events and remains clinically stable nearly one year postoperatively.

## Discussion

This case illustrates the diagnostic challenge of distinguishing a true LAAT from echodense mimics in a patient with rheumatic mitral stenosis and atrial fibrillation. TEE offers high sensitivity and specificity for LAAT detection, but accuracy may be reduced by artifacts, two-dimensional imaging limitations, and normal anatomical variants such as the Coumadin ridge or pectinate muscle trabeculations. In this patient, preoperative TEE demonstrated an echodense LAA mass concerning for thrombus, yet no thrombus was found intraoperatively, highlighting the potential for false-positive results.

Accuracy and limitations of TEE

TEE has reported sensitivity and specificity rates of 83-97% for LAAT diagnosis, with a meta-analysis by Aimo et al. showing pooled values of 97% (95% CI, 77-100%) and 94% (95% CI, 87-98%), respectively [5-7]. Specificity can be lowered by spontaneous echo contrast (SEC) and complex LAA anatomy, which may lead to unnecessary interventions such as surgical LAA ligation or prolonged anticoagulation. 

Adjunct imaging modalities 

Ultrasound-enhancing agents (UEAs), composed of microbubbles that improve endocardial definition, can aid in differentiating thrombus from artifacts, anatomic structures, and SEC when TEE images are inconclusive. While FDA-approved only for left ventricular opacification, off-label cardiovascular uses have shown value. In one study of 100 TEE patients, UEA administration improved LAA visualization and increased diagnostic confidence when excluding thrombus before cardioversion [8,9].

LAA ejection flow velocity measurement is another tool for thromboembolic risk assessment, independent of rhythm. Velocities <40 cm/s are linked to increased stroke risk and SEC, with thresholds ≤35 cm/s associated with higher prevalence of thrombus [10,11].

Three-dimensional (3D) TEE can improve diagnostic certainty by reducing equivocal findings on two-dimensional (2D) TEE. In a study of 104 patients, 3D TEE enabled a definitive diagnosis in 99% of cases versus 88.5% with 2D TEE [12]. Cardiac CT is another reliable alternative, particularly when TEE is contraindicated or inconclusive [13]. In the same 3D TEE study, cardiac CT clarified three equivocal cases, excluding LAAT in one and confirming LAA sludge in two. Early-phase cardiac CT demonstrates a specificity of 0.89 (95% CI, 0.85-0.92) for the detection of LAAT, while delayed-phase cardiac CT shows a specificity of 1.00 (95% CI, 0.98-1.00) [14].

In this case, severe SEC would likely have yielded a low LAA velocity; UEA could have improved visualization, and CT could have definitively excluded thrombus. Because surgical intervention was already indicated, these additional studies were unlikely to alter management, and the associated costs were not justified. 

Limitations and future directions

A prior embolic event cannot be entirely excluded, and the lack of adjunct imaging limits definitive assessment of the suspected mass. Future research should focus on evidence-based imaging algorithms and standardized protocols for patients with rheumatic mitral stenosis and atrial fibrillation when TEE findings are equivocal, balancing diagnostic certainty against cost and procedural risk. 

## Conclusions

This case highlights the limitations of relying solely on TEE for diagnosing LAAT, particularly in patients with rheumatic mitral stenosis with or without chronic atrial fibrillation, a population with an increased prevalence of left atrial thrombus and spontaneous echocardiographic contrast that may complicate echocardiographic interpretation. Based on the intraoperative findings and overall clinical context, we believe the observed “mass” most likely represented transient sludge rather than a reverberation artifact.

Although this report represents a single case and therefore has limited generalizability, it underscores the broader value of a multimodal approach. While additional imaging did not alter management in this patient due to the indication for mitral valve replacement, adjunctive modalities, such as ultrasound-enhancing agents, 3D TEE, LAA flow velocity assessment, and cardiac CT, should be considered when TEE findings are equivocal, and the results could influence surgical planning or anticoagulation strategy.
